# Drug-Drug Interaction Study of Benznidazole and E1224 in Healthy Male Volunteers

**DOI:** 10.1128/AAC.02150-19

**Published:** 2021-03-18

**Authors:** Isabela Ribeiro, Bethania Blum, Jayme Fernandes, Glaucia Santina, Makoto Asada, Michael Everson, Edgar Schuck, Ethel Feleder, Eric Evene, Virginie Gualano

**Affiliations:** a Drugs for Neglected Diseases initiative (DNDi), Geneva, Switzerland; b Drugs for Neglected Diseases initiative (DNDi), Rio de Janeiro, Brazil; c Eisai Co., Ltd., Tokyo, Japan; d Eisai, Inc., Woodcliff Lake, New Jersey, USA; e F.P. Clinical Pharma, Buenos Aires, Argentina; f PhinC Development, Massy, France

**Keywords:** E1224, benznidazole, Chagas disease, *Trypanosoma cruzi*, pharmacokinetics

## Abstract

E1224 is a prodrug of ravuconazole (RVZ), an antifungal drug with promising anti-Trypanosoma cruzi activity, the causative organism of Chagas disease (CD). This study was designed to assess the pharmacokinetics (PK) and safety interactions of benznidazole (BNZ), the drug of choice for treatment of CD, and E1224 in healthy volunteers. This open-label, single-center, sequential, single- and multiple-oral-dose study enrolled 28 healthy male subjects. These subjects received BNZ (2.5 mg/kg) once daily on days 1 and 9 and twice daily from day 12 to day 15 and E1224 once daily from day 4 to day 15 (loading dose of 400 mg for 3 days and maintenance dose of 100 mg for 9 days). The maximum concentration (*C*_max_) and area under the concentration curve from zero to infinity for BNZ were comparable, whether BNZ was given alone or with E1224 at steady state, with ratios of geometric means for BNZ-RVZ to BNZ of 0.96 and 0.83 and corresponding 90% confidence intervals (CIs) of 0.91 to 1.10 and 0.80 to 0.87, respectively. However, RVZ *C*_max_ and area under the concentration curve from zero to 24 h increased by about 35% when concomitantly administered with BNZ at steady state (ratio of geometric means for RVZ-BNZ/RVZ of 1.31 and 1.36 and corresponding 90% CIs of 1.23 to 1.39 and 1.31 to 1.41, respectively). Both compounds were well tolerated. There were no clinically relevant safety interactions between E1224 and BZN. Given these results, coadministration of RVZ and BNZ should not require any adaptation of E1224 dosing.

## Abstract (precis)

E1224 is a prodrug of ravuconazole (RVZ), an antifungal drug with promising anti-Trypanosoma cruzi activity, the causative organism of Chagas disease (CD). This study was designed to assess the pharmacokinetics (PK) and safety interactions of benznidazole (BNZ), the drug of choice for treatment of CD, and E1224 in healthy volunteers.

## INTRODUCTION

Chagas disease is an important global neglected tropical disease caused by Trypanosoma cruzi. Significant mortality and morbidity are observed in 20 to 30% of those chronically affected, with development of cardiac and digestive organ involvement decades after initial infection. Treatment is mainly based on benznidazole (BNZ) and nifurtimox, two nitroaromatic compounds developed in the 1960s and 1970s (1–3).

BNZ, a nitroimidazole derivative, is the drug of choice for treating adults and children with Chagas disease in most countries. After a single administration of 100 mg, the time to maximum plasma concentration (*t*_max_) is about 3.5 h and the maximum plasma concentration (*C*_max_) is between 2.2 and 2.6 μg/ml (4). The BNZ half-life (*t*_1/2_) is about 12 h (ranging from 10.5 to 13.6 h) (5). The effects of food and drug-drug interactions have not been studied, and little is known about the metabolism and absorption of BNZ; therefore, its interaction with other drugs cannot be easily anticipated. BNZ metabolism is likely to occur via a cytochrome P450 (CYP); CYP1A2, CYP2E1, and CYP2A6 are suspected, but the isoenzyme responsible has not yet been clearly identified (6, 7).

This treatment is constrained by safety and tolerability (8, 9) issues, and there is limited robust data on efficacy in chronic disease (7). BNZ is poorly tolerated, frequently resulting in an interruption of treatment (7–9). New, better-tolerated therapeutic options and new treatment regimens are needed.

The prodrug (BF) E1224 is a monolysine form of ravuconazole (RVZ), an inhibitor of ergosterol biosynthesis, more specifically a C-14 α-demethylase inhibitor. E1224 and ravuconazole showed promising *in vitro* and *in vivo* activities against T. cruzi (10–12). The MICs of ravuconazole for T. cruzi epimastigotes and amastigotes were 300 nmol/liter (221 ng/ml) and 1 nmol/liter [7.4 ng/ml]), respectively (11). No *in vitro* studies have been performed to evaluate the antiprotozoal activity of E1224 itself, as the latter is readily and completely converted into ravuconazole by plasma dephosphorylase enzymes (e.g., alkaline phosphatase) and would not be expected to demonstrate any intrinsic antiprotozoal activity in assays without metabolic enzymes. E1224 was shown to be curative in murine models of T. cruzi infection, with 71.5% (at 20 and 40 mg/kg of body weight), 85.7% (at 30 and 50 mg/kg), and 100% (at 10 mg/kg) of animals cured at the end of a 6-month follow-up period, versus 87.5% for benznidazole at 100 mg/kg (12). Following single oral administration of E1224, plasma concentrations of RVZ attain peak levels at approximately 2 to 4 h. RVZ has a long *t*_1/2_, ranging from a mean of 157 h to 221 h (6.6 to 9.2 days) (13). *C*_max_ and area under the concentration curve (AUC) values of RVZ in the plasma increase almost dose-proportionally. After multiple oral dosings, RVZ accumulates over time, but with selection of loading and maintenance doses, it is possible to attain steady state on day 7. Based on *in vitro* and *in vivo* assays, E1224 appears to have a moderate inhibitory effect on CYP3A4, but it showed no clinically important effect on the metabolism of probe drugs mediated by CYP2C19, CYP2D6, and CYP1A2 and no effect on the metabolism of drugs mediated by CYP2C9 (14).

In human studies, E1224 and another ergosterol biosynthesis inhibitor, posaconazole, demonstrated a rapid, but transient suppressive effect on parasite clearance, while BZN showed early and sustained efficacy until 12 months (13, 15). Model-based calculations with the maximum observed average concentrations show that if the E1224 dose was increased and treatment duration prolonged to, e.g., 12 weeks, the probability of relapse would fall below 20%. Since one of the main limitations of BNZ treatment relates to safety, tolerability, and the long duration of treatment, it was hypothesized that combination treatment regimens could potentially impact overall treatment effectiveness and compliance. In addition, depending on the choice of regimen, combination therapy could be used to improve efficacy and potentially reduce the risk of resistance to BNZ (12). *In vivo* combination studies in a murine model demonstrated that concomitant treatment with E1224 and benznidazole was more effective at reducing circulating parasite levels and inducing parasitological cure than either of the drugs given alone, even against highly drug-resistant organisms (12). BNZ is at least partially metabolized by CYP3A4, and a weak inhibition of BNZ metabolism could therefore be expected with E1224. As no relevant *in vivo* or *in vitro* data were available and since BNZ and E1224 are intended to be administered concomitantly in patients with Chagas disease, an *in vivo* interaction study in healthy subjects was needed. This interaction study in healthy volunteers was designed to assess the pharmacokinetic (PK) interaction of BNZ with E1224.

Upon review of the safety and tolerability profiles of these two drugs, the key safety concern was the potential of concomitant administration of these compounds to cause drug-induced liver injury. Indeed, both E1224 and BNZ cause dose-related increases in serum markers (11, 13) for hepatocellular injury (alanine aminotransferase [ALAT] and aspartate aminotransferase [ASAT]) in a subset of treated patients. Elevations may be consistent with observations with other drugs in their respective classes. It was, therefore, important to examine the impact of interactions between the two compounds BNZ and E1224 on safety.

## RESULTS

The very long half-life of RVZ (more than 200 h) did not allow a classical randomized crossover design with two successive periods of treatment. With the design chosen, it was possible to study the effect of E1224 on the PK disposition of BNZ and the effect of BNZ on the PK disposition of E1224. The effect on E1224 was studied at steady state, and the effect on BNZ was studied after a single dose.

### Subject disposition.

Between November and December 2014, a total of 28 healthy male subjects were randomized at one study site in Argentina. All the subjects received BNZ at 2.5 mg/kg of body weight once a day on days 1 and 9 and then at 2.5 mg/kg twice a day (12 h apart) from day 12 to day 15 and received E1224 once a day from day 4 to day 15, with a loading dose of 400 mg once daily from day 4 to day 6, followed by a maintenance dose of 100 mg once daily from day 7 to day 15. All subjects completed the study.

### Baseline characteristics.

Subjects ranged in age from 18 to 45 years (mean, 29 years) and had a body mass index (BMI) of 18.3 to 28.0 kg/m^2^ (Table 1). Some minor abnormalities were reported in baseline levels of laboratory (biochemistry and hematology) parameters. Three study subjects had ALAT levels above the normal upper limit, with values between 41 and 53 IU/liter, for a normal upper limit of <40 IU/liter, while only one subject had elevated ASAT, with a value of 69 IU/liter for a normal upper limit of <40 IU/liter.

**TABLE 1:** Demographic data

Parameter^*a*^	Result for all subjects (*n* = 28)	
	Mean (SD)	Range
Age, yr	29.00 (7.93)	18.00–45.00
Ht, cm	172.36 (6.98)	161.00–188.00
Wt, kg	73.55 (10.49)	55.00–94.50
BMI, kg/m²	24.72 (2.95)	18.30–27.97

a BMI, body mass index.

### Pharmacokinetics.

**FIG 1:**
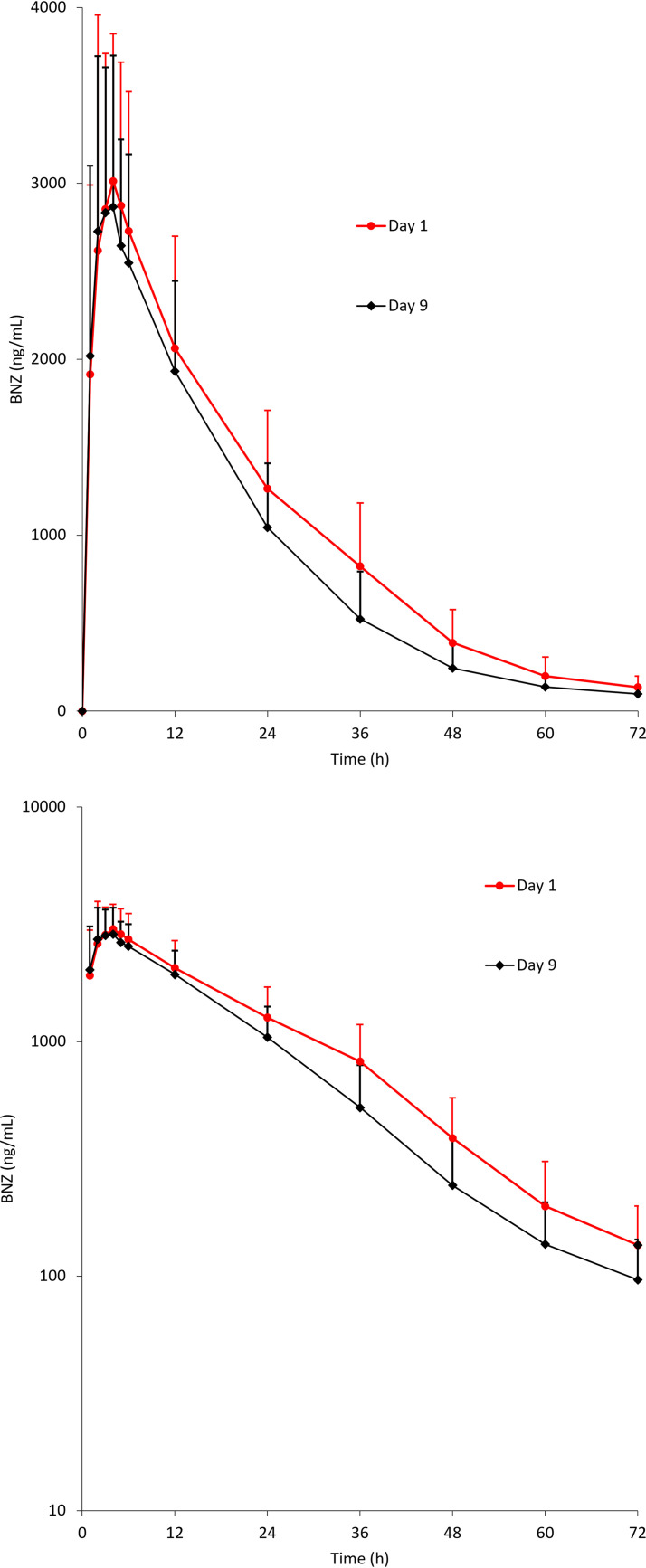
Mean + standard deviation benznidazole (BNZ) blood concentrations versus time after single-oral-dose administration of 2.5 mg/kg BNZ on days 1 (BNZ alone) and 9 (BNZ plus ravuconazole). (Top graph) Untransformed scale. (Bottom graph) Semi-log scale.

The mean BNZ concentration-time profiles after BNZ administration in the absence or presence of E1224 are shown in Fig. 1. The summary statistics for BNZ PK parameters are shown in Table 2. Following administration of BNZ with E1224, the geometric mean (GM) of the BNZ *C*_max_ decreased by 4% (3,221 ng/ml versus 3,364 ng/ml), and the GM of the area under the concentration-time curve from time zero to infinity (AUC_0–∞_) and AUC from time zero to the last quantifiable concentration (AUC_0–_*_t_*) decreased by 16% (61,162 and 59,299 ng · h/ml, respectively, versus 72,720 and 70,559 ng · h/ml). The median *t*_max_ values were similar under both conditions (3 h). The GM values of *t*_1/2_ were also comparable when BNZ was administered alone or with E1224 (12 versus 10 h). Formal statistical analyses supported the above observations since the 90% confidence intervals (CIs) for *C*_max_ (0.91 to 1.10) and AUC_0–∞_ (0.80 to 0.87) were within or very close to the reference range for bioequivalence (0.80 to 1.25) (16). Corresponding point estimates were 0.96 for *C*_max_ and 0.83 for AUC_0–∞_.

**TABLE 2:** Benznidazole pharmacokinetic parameters after single-oral-dose administration of 2.5 mg/kg BNZ alone or with E1224

Treatment^*a*^	Result for parameter^*b*^:				
	*C*_max_, ng/ml	AUC_0–_*_t_*, ng · **h/ml**	AUC_0–∞_, ng · **h/ml**	*t*_1/2_, h	*t*_max_, h
BNZ (*n* = 28)	GM, 3,364 (CV%, 26.4)	GM, 70,559 (CV%, 32.0)	GM, 72,720 (CV%, 32.7)	GM, 12.2 (CV%, 25.2)	Median, 3.0 (range, 1.0–6.0)
BNZ + E1224 (*n* = 28)^*c*^	GM, 3,221 (CV%, 25.7)	GM, 59,299 (CV%, 29.8)	GM, 61,162 (CV%, 29.8)	GM, 10.0 (CV%, 19.4)	Median, 3.0 (range, 1.0–6.0)
BNZ + E1224 vs BNZ	PE, 0.96 (90% CI, 0.91–1.10)	PE, 084 (90% CI, 0.81–0.88)	PE, 0.83 (90% CI, 0.80–0.87)		

a BNZ, benznidazole.

b *C*_max_, maximum concentration; AUC_0–_*_t_*, area under the curve from time zero to the last measurement; AUC_0–∞_, area under the curve from time zero to infinity; *t*_1/2_, terminal half-life; *t*_max_, time of *C*_max_; GM, geometric mean; CV%, coefficient of variation; PE, point estimate; CI, confidence interval.

c *n* = 28, except for AUC_0–∞_ and *t*_1/2_, where *n* = 27.

**FIG 2:**
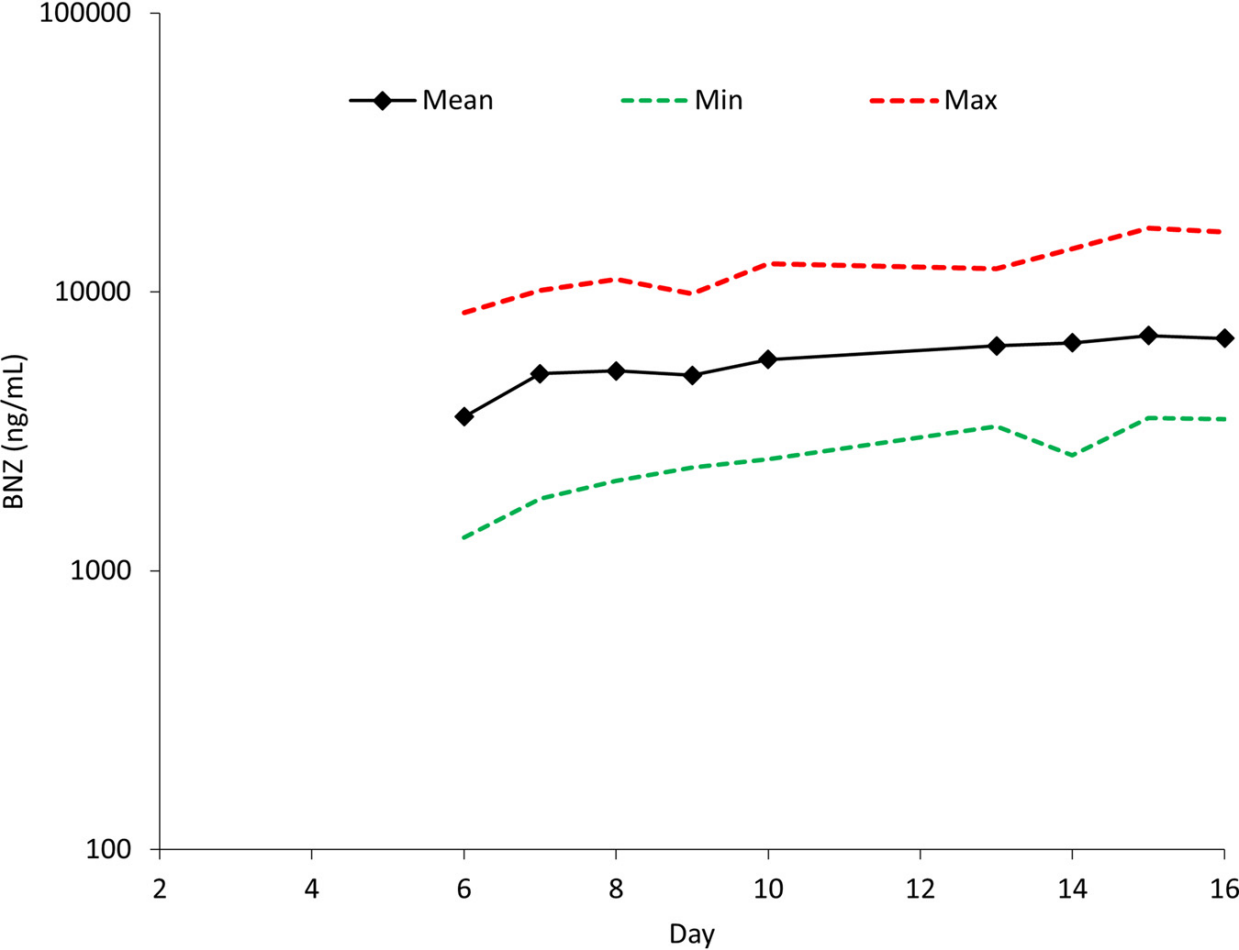
Mean, minimum (Min), and maximum (Max) predose concentrations of ravuconazole (RVZ) from day 6 to day 16 (i.e., from 2 to 12 days of administration).

**FIG 3:**
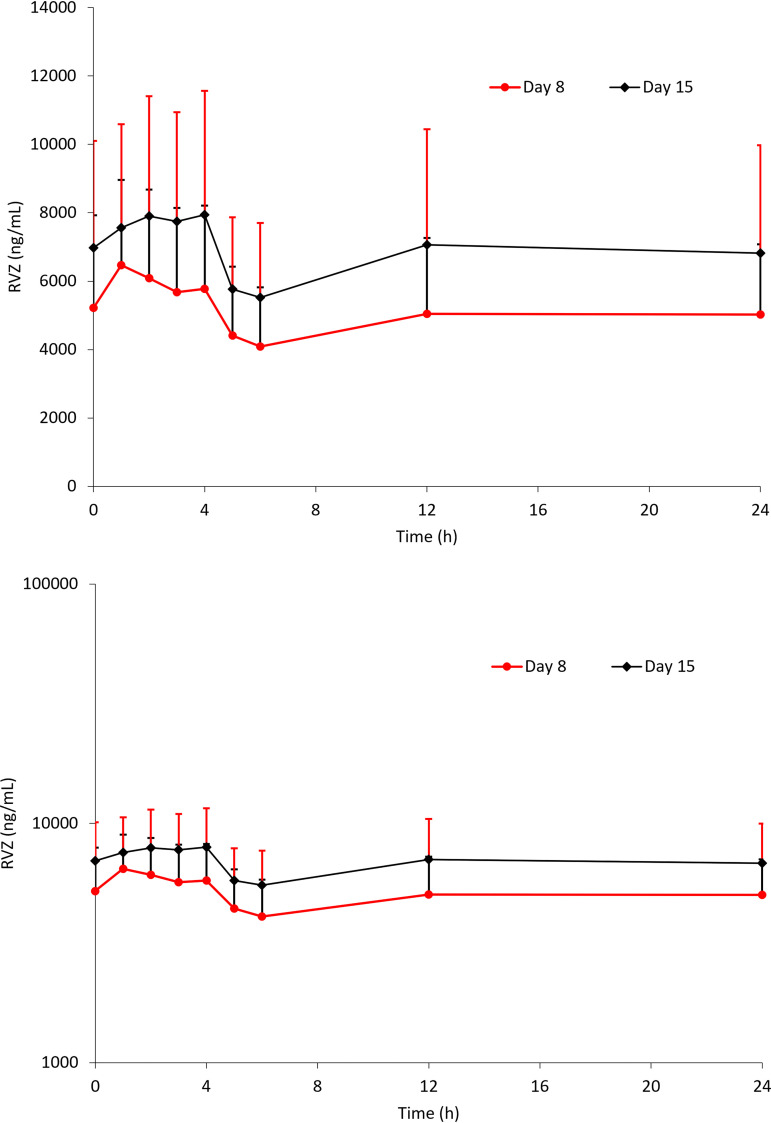
Mean + standard deviation ravuconazole (RVZ) blood concentrations versus time on days 8 (RVZ alone) and 15 (RVZ + benznidazole). (Top graph) Untransformed scale. (Bottom graph) Semi-log scale.

Following multiple-dose administration of E1224, the predose concentration of RVZ from day 6 to day 16 (i.e., from 2 to 12 days of administration) is presented in Fig. 2. Mean (standard deviation [SD]) predose RVZ blood concentrations were 5,217 (2,707) ng/ml and 6,976 (3,127) ng/ml on days 8 and 15, respectively. The mean RVZ concentration-time profiles at steady state after E1224 administration in the absence or presence of BNZ are shown in Fig. 3. The summary statistics for RVZ PK parameters are shown in Table 3.

**TABLE 3:** Ravuconazole pharmacokinetic parameters after oral administration of E1224 alone or with 2.5 mg/kg benznidazole at steady state

Treatment^*a*^	Result for parameter^*b*^:		
	*C*_max_, ng/ml	AUC_0–24_, ng · **h/ml**	*t*_max_, h
E1224 (*n* = 28)	GM, 6,366 (CV%, 38.5)	GM, 111,851 (CV%, 41.3)	Median, 1.0 (range, 0.0–12.0)
BNZ + E1224 (*n* = 28)	GM, 83,286 (CV%, 43.7)	GM, 151,658 (CV%, 43.2)	Median, 3.0 (range, 1.0–12.0)
BNZ + E1224 vs E1224	PE, 1.31 (90% CI, 1.23–1.39)	PE, 1.36 (90% CI, 1.31–1.41)	

a BNZ, benznidazole.

b *C*_max_, maximum concentration; AUC_0–24_, area under the curve from time zero to 24 h; *t*_max_, time of *C*_max_; GM, geometric mean; CV%, coefficient of variation; PE, point estimate; CI, confidence interval.

Following administration of E1224 with BNZ, the GM of RVZ *C*_max_ increased by 31% (8,328 versus 6,366 ng/ml), and the GM of AUC from time zero to 24 h postdose (AUC_0–24_) increased by 36% (151,658 versus 111,851 ng · h/ml). The median RVZ *t*_max_ was delayed after administration with BNZ (3 h versus 1 h). Formal statistical analyses showed that the 90% CIs for *C*_max_ (1.23 to 1.39) and AUC_0–24_ (1.31 to 1.41) were not fully included in the reference range for bioequivalence (0.80 to 1.25) (16). The relative bioavailability of RVZ given concomitantly with BNZ resulted in an increase of about 35%, given the *C*_max_ and AUC_0–24_ point estimates of 1.31 and 1.36, respectively.

### Safety and tolerability.

No severe or serious adverse events were reported. A total of 13 drug-related treatment emergent adverse events (TEAEs) were reported by 9 out of 28 subjects. TEAEs were reported on days when E1224 was administered alone (3 TEAEs) or coadministered with BNZ (10 TEAEs). All of the reported events were of mild or moderate intensity. The most frequent TEAEs consisted of skin disorders (4 cases of rash, 1 case of pruritus, and 1 case of erythema). Other TEAEs were laboratory result abnormalities (1 case of ASAT increase, 2 cases of non-clinically significant bilirubin increase, and 2 cases of eosinophilia) and headache (2 cases). No subject discontinued treatment due to TEAE.

In electrocardiograms (ECG) or vital signs, only incidental, nonspecific abnormalities were reported.

## DISCUSSION

E1224 is in development for the treatment of mycetoma and Chagas disease and is being investigated for use in combination with other treatments to improve efficacy. As BNZ is the current standard of care for Chagas disease, E1224 and BNZ are under consideration for concomitant administration.

No *in vitro* data were available on potential safety interactions. Evaluation of BNZ-ravuconazole interaction *in vitro* demonstrated a simple additive effect, based on the mean fractional inhibitory concentrations (ΣFICs) of 0.76 to 0.98 for the Y strain and 1.10 to 1.56 for the Colombian strain (12). As previously discussed, follow-up investigations of the E1224 and BNZ combination in murine models of T. cruzi (12) showed a positive interaction between E1224 and BNZ in the treatment of early infections by a multidrug-resistant strain, with a 100% cure rate and reductions in the period of patent parasitemia. It was considered necessary to investigate the effect of coadministering the two drugs on PK. Due to the very long half-life of RVZ, a sequential design was chosen to assess the effect of E1224 on the PK of BNZ and the effect of BNZ on the PK of RVZ. This design was consistent with guidelines for drug interaction studies (17, 18). In this study, the effect of the interaction on E1224 was studied at steady state, and the effect on BNZ was studied after a single dose in healthy subjects. Following administration of BNZ at dose of 2.5 mg/kg, BNZ PK parameters (*C*_max_, 3,364 ng/ml; *t*_max_, 3 h; *t*_1/2_, 12 h) were consistent with the results of previously published studies (4). When administered with E1224, a limited decrease in BNZ exposure was observed, with a decrease in *C*_max_ of 4% and a decrease in AUC_0–∞_ and AUC_0–_*_t_* of 16%. Other parameters (*t*_max_ and *t*_1/2_) were comparable for both conditions. The decrease in exposure was not clinically significant, with the 90% CIs for *C*_max_ and AUC_0–∞_ within the reference range for bioequivalence. These results indicated that RVZ had no impact on the PK characteristics of single-oral-dose administration of 2.5 mg/kg BNZ.

At steady state, following concomitant administration of prodrug E1224 with BNZ, overall RVZ exposure significantly increased by about 35%, with the 90% CIs for *C*_max_ and AUC_0–24_ slightly exceeding the reference range.

No clinically relevant safety interactions were documented from the perspective of safety. The skin disorders, in particular rash, that occurred after the first administration of E1224 with BNZ are consistent with common adverse events (AEs) observed for BNZ and previously reported for E1224 (6, 7, 9, 13). Other AEs were in line with those previously reported following administration of E1224 (13). Under the conditions of this study, safety and tolerability may be considered to be good, and no clinically relevant safety interactions were documented between BNZ and E1224.

### Conclusions.

Given the lack of impact of RVZ on BNZ PK and the significant 35% decrease in exposure of RVZ with BNZ, which was not anticipated to have clinical significance, it appears that coadministration of RVZ and BNZ does not require any adaptation of the E1224 dose. Concomitant administration of BNZ and E1224 was well tolerated.

## MATERIALS AND METHODS

### Study population.

Male subjects in good health on the basis of medical history, physical examination, vital signs, ECG, and routine laboratory safety tests, of ages from 18 to 45 years, with BMIs of 18 to 28 kg/m^2^, and nonsmokers or light smokers (≤5 cigarettes per day) were eligible.

The study was conducted in accordance with Good Clinical Practices and the ethical principles of the Declaration of Helsinki. The study was approved by an independent Ethics Committee in Argentina before the study start. All subjects provided written informed consent prior to participation. The subjects were enrolled from 7 November 2014 (first subject screening visit) to 13 December 2014 (last subject last visit).

### Study design.

This phase I trial was an open-label, single-center, sequential single- and multiple-oral-dose drug-drug interaction study in healthy male subjects. A screening visit was performed within the 3 weeks preceding treatment initiation. Subjects were hospitalized from the evening of day 1 to the morning of day 2. Thereafter, ambulatory visits were planned on days 2 (evening), 3 (morning and evening), and 4 (morning) and then on the mornings of days 5 to 7. Subjects were hospitalized from the evening of day 7 to the morning of day 10 for a second in-house period. Ambulatory visits were planned from the evening of day 10 to the morning of day 14. Subjects were then hospitalized for the third hospitalization from the evening of day 14 to day 16. Finally, the end-of-study (EOS) visit was performed between 5 and 7 days after the last drug administration.

### Dose administered.

The therapeutic dose of BNZ was administered: i.e., 5 mg/kg divided in two doses given with an interval of 12 h, corresponding to 2.5 mg/kg twice daily.

A multiple-dose regimen was chosen for E1224 (a prodrug for the active ingredient RVZ). Subjects received a loading dose of 400 mg once daily for 3 days, followed by a maintenance dose of 100 mg once daily for 9 days, to reach steady state as soon as possible and to reduce the risk of liver enzyme increase.

**FIG 4:**
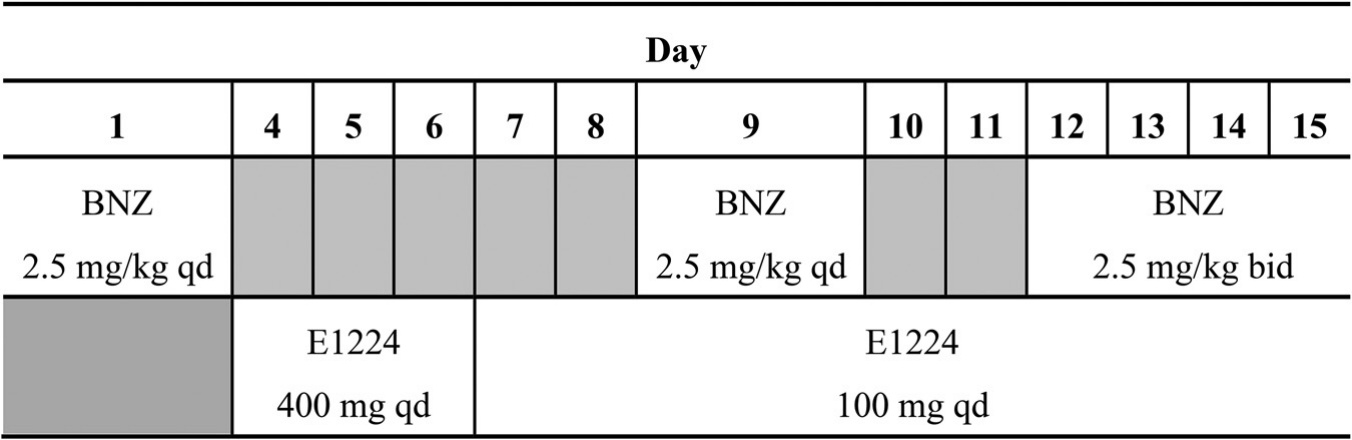
Scheme of administration. BNZ, benznidazole; qd, once a day; bid, twice a day.

BNZ and E1224 were administered to all subjects under fasted conditions according to the schema in Fig. 4.

### Study assessments.

Safety assessments, including adverse events, clinical examination, vital signs, ECGs, clinical laboratory parameters, and urinalysis, were performed at defined time points from screening to EOS.

The safety reporting period for this trial began upon subject enrollment in the trial (after signature of informed consent) and ended at 4 weeks after the last dose of study medications. Investigators were required to report all directly observed TEAEs and all TEAEs spontaneously reported by the trial subject. In addition, each trial subject was questioned about the occurrence of AEs in all study visits.

Information on AEs has to be evaluated by a physician at each patient visit. Each AE was classified by the investigator as serious or nonserious and regarding possible relationship to the study medication. AE intensity was graded using the National Cancer Institute’s Common Terminology Criteria for Adverse Events (CTCAE; version 4.03).

Clinical laboratory tests were performed during the study, including complete blood count (hemoglobin, red blood cell, hematocrit, mean corpuscular volume, mean corpuscular hemoglobin, mean cell hemoglobin concentration, and white blood cell including differential and platelet counts), clinical chemistry (alkaline phosphatase [ALP], ALAT, ASAT, γ -glutamyl transferase [GGT], total bilirubin, total protein, creatinine, fasting glucose, albumin, urea, electrolytes (Na^+^, K^+^, and Cl^−^), alcohol test, and urine dipstick analysis. Assessments were performed at screening and at the day 1, 4, 7, 9, 10, 12, 13, 14, and 15 and EOS visits.

ECGs were performed at screening, visit days 4, 7, and 12, and at the end of study. Blood samples were collected for BNZ and RVZ PK analyses. For BNZ, blood samples were collected on days 1 and 9 predose and at 1, 2, 3, 4, 5, 6, 12, 24, 36, 48, 60, and 72 h postdose. For RVZ, PK samples were collected on days 6, 7, 13, and 14 predose, on days 8 and 15 predose, and at 1, 2, 3, 4, 5, 6, 12, and 24 h postdose.

### Bioanalysis.

A liquid chromatography method with tandem mass spectrometry was developed, validated, and used to analyze BNZ and RVZ dry blood spots in the laboratories of B & S Inovações (Engenho do Meio, Recife, Brazil) in accordance with Good Laboratory Practice. The limits of quantification of these methods were 20 ng/ml for RVZ and 50 ng/ml for BNZ.

### Pharmacokinetic analysis.

PK parameters were determined by noncompartmental analysis using Phoenix WinNonlin (Pharsight, Mountain View, CA). For BNZ, the following PK parameters were derived on days 1 and 9: *C*_max_, *t*_max_, terminal rate constant estimated by log-linear regression analysis (*k*_e_), AUC_0–_*_t_* using a linear trapezoidal method, AUC_0–∞_, and *t*_1/2_. For RVZ, the following PK parameters were derived on days 8 and 15: *C*_max_, *t*_max_, and AUC_0–24_ using a linear trapezoidal method.

### Sample size estimation.

The sample size was determined in order to achieve a power of at least of 80% at a type I error (α) of 5%, in the case of reference bioequivalence margins (0.80 to 1.25), as recommended by U.S. Food and Drug Administration guidance documents (16), and an intraindividual variability of 25%. A total of 28 subjects was deemed sufficient to demonstrate a lack of interaction between the two drugs.

### Statistical analysis.

All subjects who received at least one dose of the study medication were enrolled in the safety analysis set. All subjects who completed the study without events likely to bias the PK evaluation were included in PK analysis set.

Descriptive statistics were performed on demographic data, vital signs (temperature, blood pressure, and heart rate), clinical laboratory parameters, ECG, and AEs.

Descriptive statistics for individual blood concentrations and PK parameters were performed with Phoenix WinNonlin 6.3 and are presented for each compound (i.e., BNZ and RVZ) by day.

Statistical analysis was performed using SAS software version 9.4. The effect of BNZ on RVZ PK parameters and vice versa was evaluated based on AUC_0–∞_, AUC_0–_*_t_*, and *C*_max_ for BNZ and AUC_0–24_ and *C*_max_ for RVZ by analysis of variance (ANOVA) using a mixed-effects model applied to log-transformed PK parameters with treatment as fixed effects and subjects as random effects. For each parameter, a point estimate for the ratio of geometric means (RVZ + BNZ [day 15]/RVZ alone [day 8]) and (RVZ + BNZ [day 9]/BNZ alone [day 1]) was obtained by calculating the difference of least square means on the logarithmic scale and subsequent back-transformation using the anti-log function. Likewise, the 90% CI for the ratios was obtained, and a point estimate for the ratio of geometric means (RVZ + BNZ [day 15]/RVZ alone [day 8]) and (RVZ + BNZ [day 9]/BNZ alone [day 1]) was obtained by least-squares means. It was concluded that there was no interaction if the 90% CI for the ratio for all PK parameters fell entirely within the reference interval of 0.80 to 1.25.

The analysis of *t*_max_ values was based on the nonparametric Wilcoxon signed-rank test.
